# “ET Plus”: Instability of the Diagnosis During Prospective Longitudinal Follow-up of Essential Tremor Cases

**DOI:** 10.3389/fneur.2021.782694

**Published:** 2021-12-16

**Authors:** Daniella Iglesias-Hernandez, Nikki Delgado, Margaret McGurn, Edward D. Huey, Stephanie Cosentino, Elan D. Louis

**Affiliations:** ^1^Department of Neurology, University of Texas Southwestern Medical Center, Dallas, TX, United States; ^2^Department of Neurology, College of Physicians and Surgeons, Columbia University, New York, NY, United States; ^3^Taub Institute for Research on Alzheimer's Disease and the Aging Brain, College of Physicians and Surgeons, Columbia University, New York, NY, United States; ^4^Department of Psychiatry, College of Physicians and Surgeons, Columbia University, New York, NY, United States

**Keywords:** essential tremor, ET plus, classification, diagnosis, clinical

## Abstract

**Background:** A recent consensus statement introduced the term “ET plus”. Although investigators have quantified the prevalence of ET plus in cross-sectional studies, patients with ET plus have not been tracked longitudinally; hence, there is no understanding of its stability over time.

**Methods:** We present prospective, longitudinal phenotypic data on an ET cohort that was followed regularly at 18-month intervals (T1, T2, T3, T4) for up to 64 months. We assigned an ET or ET plus diagnosis to each case at each time interval.

**Results:** There were 201 participants at baseline. The proportion with ET plus increased from 58.7% at baseline to 72.1% at T4 (*p* = 0.046). Of 172 (85.6%) who received a diagnosis of ET plus at one or more time intervals, the diagnosis was unstable (e.g., with reversion) in 62 (36.0%). We also assessed the stability of the clinical features of ET plus. Rest tremor was the most unstable clinical feature of ET plus; it was present in 59 participants, among whom it reverted from present to absent in 23 (39.0%). By contrast, for “memory impairment” (i.e., either mild cognitive impairment or dementia), the proportion who reverted from present to absent was only 21.3%.

**Conclusion:** These data support our two *a priori* hypotheses: (1) the prevalence of ET plus would increase progressively, as it likely represents a more advanced stage of ET, and (2) the ET plus diagnosis would not be stable over time, as cases would fluctuate with respect to their phenotypic features and their assigned diagnoses.

## Introduction

Essential tremor (ET) is a movement disorder whose conceptualization has evolved over time (1). Its initial description in the nineteenth century centered on tremor, yet ET is now recognized as a disease or family of diseases that is associated with a myriad of additional neurological features including non-motor symptoms and signs, as well as other motor signs besides tremor (e.g., balance impairment) (1–3). In response to a growing appreciation of the broad and heterogeneous distribution of these additional features across ET patients, a recent consensus statement introduced the term “ET plus” to the narrative (4). ET plus was defined as ET in the presence of additional “soft neurological” signs of uncertain significance, such as impaired tandem gait, questionable dystonic posturing, memory impairment, and other mild features that do not suffice for an alternate tremor classification (4). The new term has generated interest and several publications have assessed the proportions with ET vs. ET plus in their cohorts (5–8). On the other hand, the new classification has also engendered criticism and controversy, with considerable concern that it represents a disease stage rather than a true disease subtype (9–13). A recent postmortem study reported that the degenerative changes in the cerebellum that have been linked to ET did not differ between ET and ET plus, suggesting that the proposed new classification is not grounded in a biological difference (14).

Although several investigators have quantified the prevalence of ET plus in cross-sectional samples of patients, those with ET plus have not been tracked longitudinally, so there is no understanding of how this designation behaves over time and whether it is stable (5, 6, 8).

In this study, we present prospective, longitudinal phenotypic data on an ET cohort that was followed for up to 64 months and to whom the designation ET vs. ET plus could be assigned. We hypothesized that (1) the prevalence of ET plus would increase progressively, as it likely represents a more advanced stage of ET, and that (2) the ET plus diagnosis would not be stable over time, as cases would fluctuate with respect to their phenotypic features and their assigned diagnoses. Both of these hypotheses, if supported, suggest that ET and ET plus may not be distinct diagnostic entities.

## Methods

### Patient Selection

The Clinical-Pathological Study of Cognitive Impairment in ET (COGNET) is an ongoing, prospective, longitudinal study that has the goals of characterizing motor and cognitive deficits in ET over time as well as neuropathological features in ET after death. Enrollment began in July 2014 and eligible participants met the following criteria: (1) ET diagnosis in the absence of additional movement disorder diagnoses, (2) willingness to complete an extensive neuropsychological battery at each interval, (3) willingness to donate their brain at the time of death, (4) minimum age of 55 years old, and (5) no previous surgical treatment for ET. Once participants were deemed eligible, the research team performed comprehensive neurological and cognitive evaluations at the following time intervals: baseline (T1), 18 months (T2), 36 months (T3), and 54 months from baseline (T4). An informed consent was obtained for every participant, and at their time of death, extensive neuropathological data were collected to complement the detailed phenotypic data.

The COGNET study enrolled 256 participants between June 2014 and March 2020. As part of the stated goal of the COGNET study, the current analyses assessed motor deficits in ET over time. For these analyses, we applied the following additional inclusion criteria: (1) participants completed at least two time intervals and (2) videotaped neurological videotaped examinations were available at each time interval. A total of 648 observations were available for ET plus reclassification: for the first and second intervals, 201 participants were included because 23 participants passed away and 32 withdrew before completing T2. During the third interval, 186 participants met criteria since 7 passed away before completing T3 and 8 had phone evaluations rather than in-person visit evaluations. In the fourth and last interval, 60 participants were included since this was the number of in-person visits available at the time.

### Phenotyping Participants

The on-site evaluation of all participants was performed by trained research assistants in participants' homes. A clinical questionnaire included demographic and clinical data, as well as age of onset of action tremor. In addition, data were collected on the presence of additional or new neurological diagnoses (e.g., Parkinson's disease [PD], dystonia, spinocerebellar ataxia, fragile X-associated tremor ataxia syndrome). The videotaped neurological examination included detailed assessments of tremor, Parkinsonism, and dystonia, which were assessed and scored by a senior movement disorders neurologist (E.D.L). Kinetic or postural tremor were rated (0–3) on 12 tasks, resulting in a total tremor score (range = 0–36, higher scores indicate more severe tremor) (15). We calculated the difference between dominant and non-dominant arm tremor scores, and used two cut-offs to define action tremor asymmetry: ≥1 point difference and ≥2 point difference (16, 17). In COGNET, rest tremor was assessed while seated (0 = none, 1 = unilaterally present, 2 = bilaterally present) and while standing (0 = none, 1 = unilaterally present, 2 = bilaterally present). The motor portion of the Unified Parkinson's Disease Rating Scale was used to assess cardinal features of parkinsonism in detail (18), although tone was not assessed in videotaped examinations. Dystonia (sustained or intermittent muscle contractions causing abnormal and often repetitive movements, postures, or both) was assessed via views of the face, neck, trunk, and extremities: while seated, standing, and walking; with posture (arms extended in front of the body and in “wing-beating” position); while drawing spirals, and while speaking and reading (19). Intention tremor was assessed during the finger-nose-finger maneuver (10 repetitions per arm) with ratings including 0 (not present), 0.5 (possibly present), and 1 (definitely present) for each arm (20). For gait assessment, individuals were asked to walk heel-to-toe in a straight line for 10 steps; the number of steps taken off a straight line was recorded.

Diagnosis of ET was confirmed by a senior movement disorders neurologist (E.D.L.) based on the participant's history and the videotaped neurological examination using the Washington Heights-Inwood Genetic Study of ET (WHIGET) diagnostic tandem criteria (15), which require moderate or greater amplitude kinetic tremor during three or more tests or head tremor in the absence of PD, dystonia, or other cause. These criteria have been shown to be reliable (21) and valid (22).

### Assigning the Cognitive Diagnosis

A neuropsychological test battery was administered by a trained research assistant, with tests that evaluated global cognition and five cognitive domains: language, memory, executive function, visuospatial ability, and attention. The Mini-Mental State Examination (MMSE) and the Montreal Cognitive Assessment (MoCA) were used as measures of global cognition (23, 24). Details have been presented in a previous publication (25). At each interval, we calculated z-scores that were normalized according to gender, age, and years of education to determine impairment in each cognitive domain. Additionally, each participant designated an informant, who provided detailed information on the participant's activities of daily living and behavior. During consensus conferences, a neuropsychologist (S.C.) and a geriatric psychiatrist (E.D.H) assigned a Clinical Dementia Rating (CDR) score and a clinical diagnosis based on the participant's test results, the informant interview, and the research assistant's impression at every interval (26). For a diagnosis of normal cognition, the CDR score could be either 0 or 0.5 with test impairment that did not meet mild cognitive impairment (MCI) criteria. An MCI diagnosis required a CDR of 0.5 in addition to impairment in two designated tests (*z*-scores ≤ −1.5). A dementia diagnosis necessitated a CDR ≥ 1 along with impaired test scores (*z*-scores ≤ −1.5) across multiple domains (2).

### Assigning ET Plus Diagnoses

We defined ET plus using the Consensus Statement, i.e., ET cases with any of the following features: memory impairment, impaired tandem gait, rest tremor, questionable dystonic posturing, or mild neurologic signs that were of unknown significance or insufficient to make an additional classification (4).

To rigorously operationalize these criteria, we made several decisions. “Memory impairment” is a characteristic that is highly prevalent at varying degrees in elderly cohorts (27), yet in the Consensus Statement, the minimum severity is not specified. To avoid near-universal application of the ET plus designation to this cohort, we set clear boundaries (diagnoses of MCI based on clinical and neuropsychological information (see above) or dementia), which provided a robust, clearly defined, reproducible metric. Second, given the high prevalence of tandem mis-steps with advanced age, we established that seven or more tandem mis-steps were required for “impaired tandem gait”. The cut-off point was based on prior data in which several hundred ET cases and age-matched controls were compared, and this cut-point established a separation point between the disease state and normal age-related gait difficulty. In the boxed table defining ET plus in the Consensus Statement, the authors wrote: “ET with tremor at rest should be classified here”. Accordingly, the presence of rest tremor in any extremity or body part was also defined as ET plus. Furthermore, we included any possible signs of parkinsonism besides rest tremor (e.g., subtle changes in arm swing, mild reduction in facial expression) that were noted in the video examination. For questionable dystonic posturing, mild, abnormal limb or neck postures (e.g., finger pointing, spooning) were included (28, 29). Intention tremor (when definitely present in both arms) was included in ET plus because in the Consensus statement, ET plus included “mild neurological signs of unknown significance” and, in that statement, intention tremor was noted to be a neurological sign that was distinct from the type of action tremor observed in ET.

Using the above metrics, we classified each individual in the cohort as either ET or ET plus at each time interval. Furthermore, in a secondary analysis, we classified each individual in the cohort but did so without including rest tremor or intention tremor as features of ET plus.

### Longitudinal Instability of ET Plus

**Figure 1:**
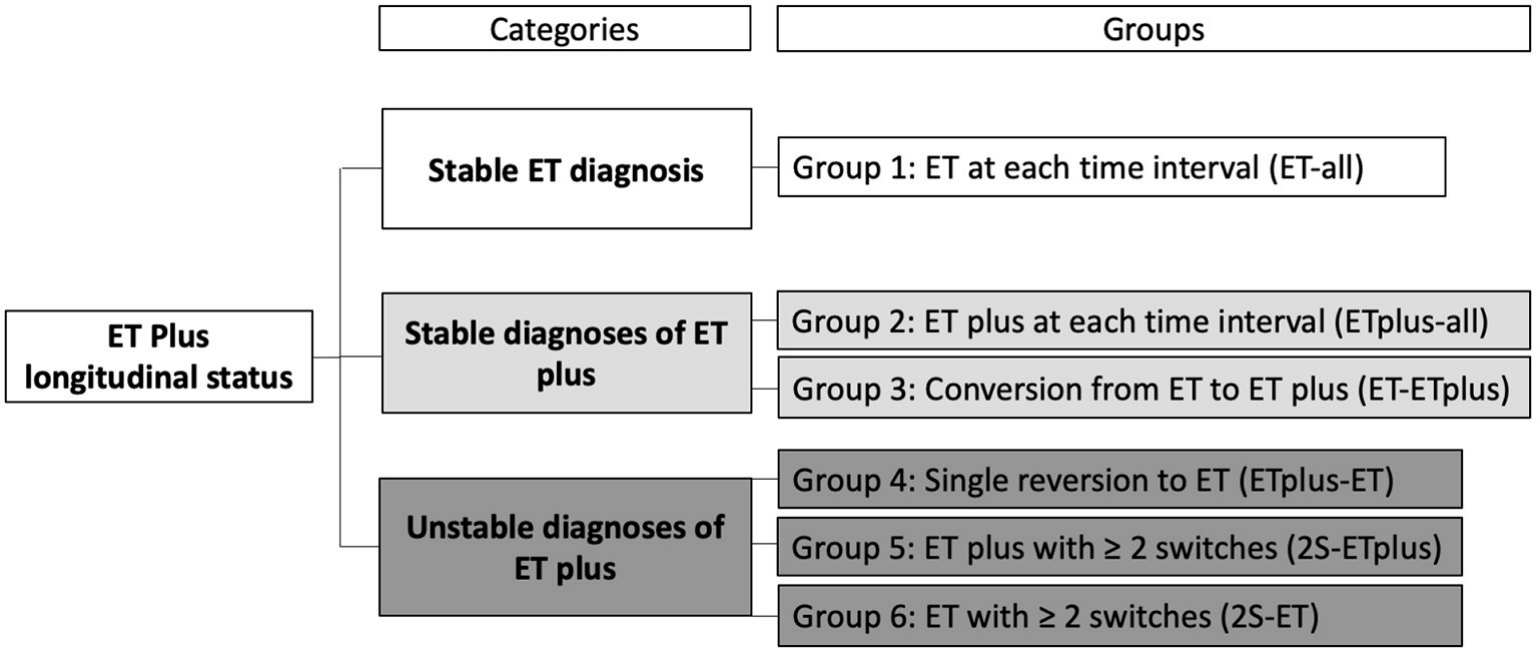
Classification of ET and ET plus diagnoses over time.

We assigned an ET or ET plus diagnosis to each case at each time interval. Based on the stability of the diagnosis over time, we developed three “diagnostic behavior over time” Categories and six “diagnostic behavior over time” Groups (Figure 1). The first Category, “Stable ET diagnosis,” included Group 1—participants labeled ET at each time interval (ET-all). The second Category, “Stable ET plus diagnosis,” included two groups: ET plus at each time interval (ET plus-all = Group 2) and conversion from ET to ET plus that then subsequently remained stable for the remaining time intervals that followed (ET-ET plus = Group 3). The third Category, “Unstable diagnoses of ET plus,” included three additional groups. Group 4 accounted for participants diagnosed as ET plus in their first interval and later had a single reversion to ET, which remained their final diagnosis (ET plus-ET). Group 5 included participants with ≥2 switches between ET and ET plus before reaching ET plus as their final diagnosis (2S-ET plus). Group 6 accounted for cases with ≥2 switches between ET and ET plus before reaching ET as final diagnosis (2S-ET).

In a similar manner, we categorized the persistence of each neurological sign. A stable neurological sign was defined as present at each time interval. By contrast, unstable neurological signs were part of the initial ET plus diagnosis but not present at every following interval.

### Statistical Analysis

**Figure 2:**
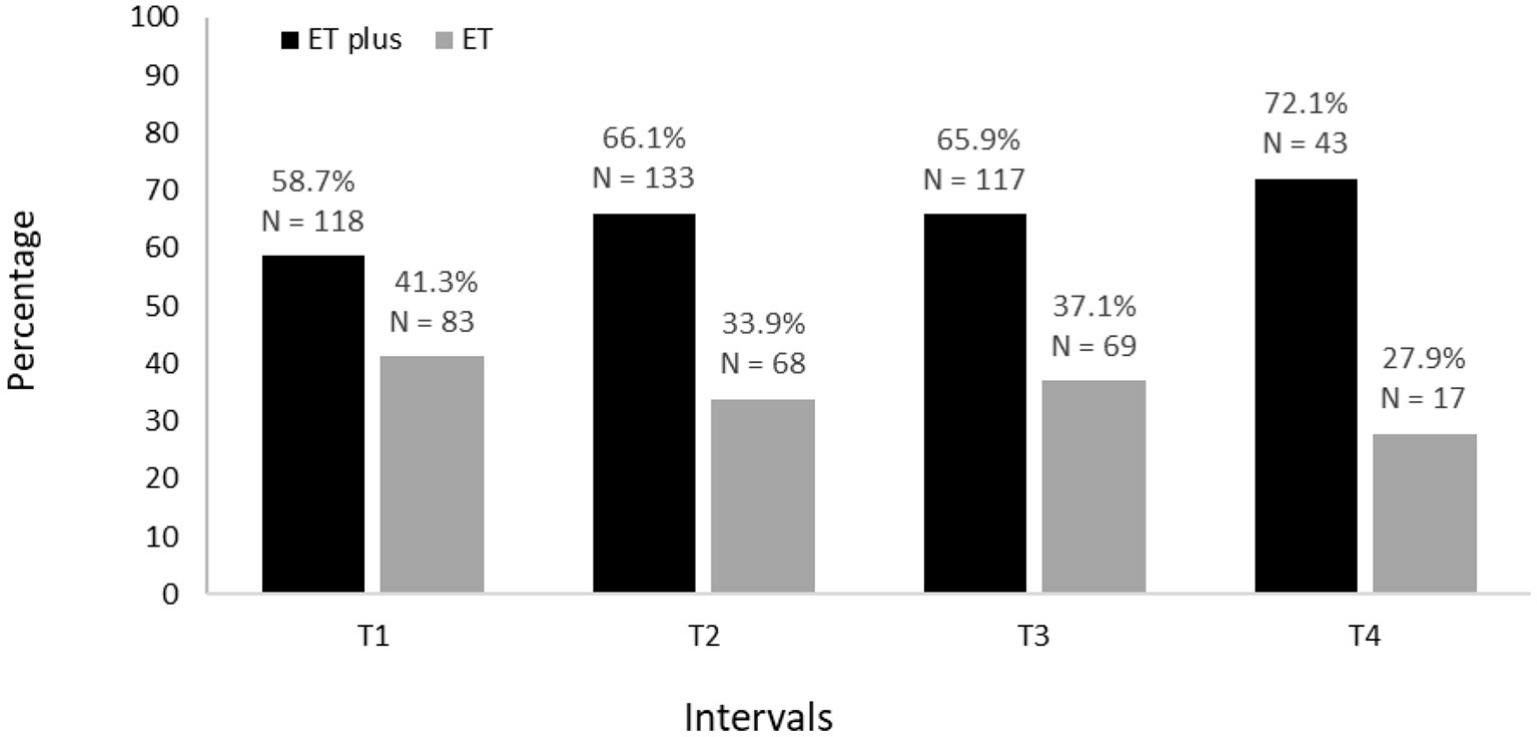
Prevalence of ET vs. ET plus from baseline (T1) to 54 months after baseline (T4).

**Figure 3:**
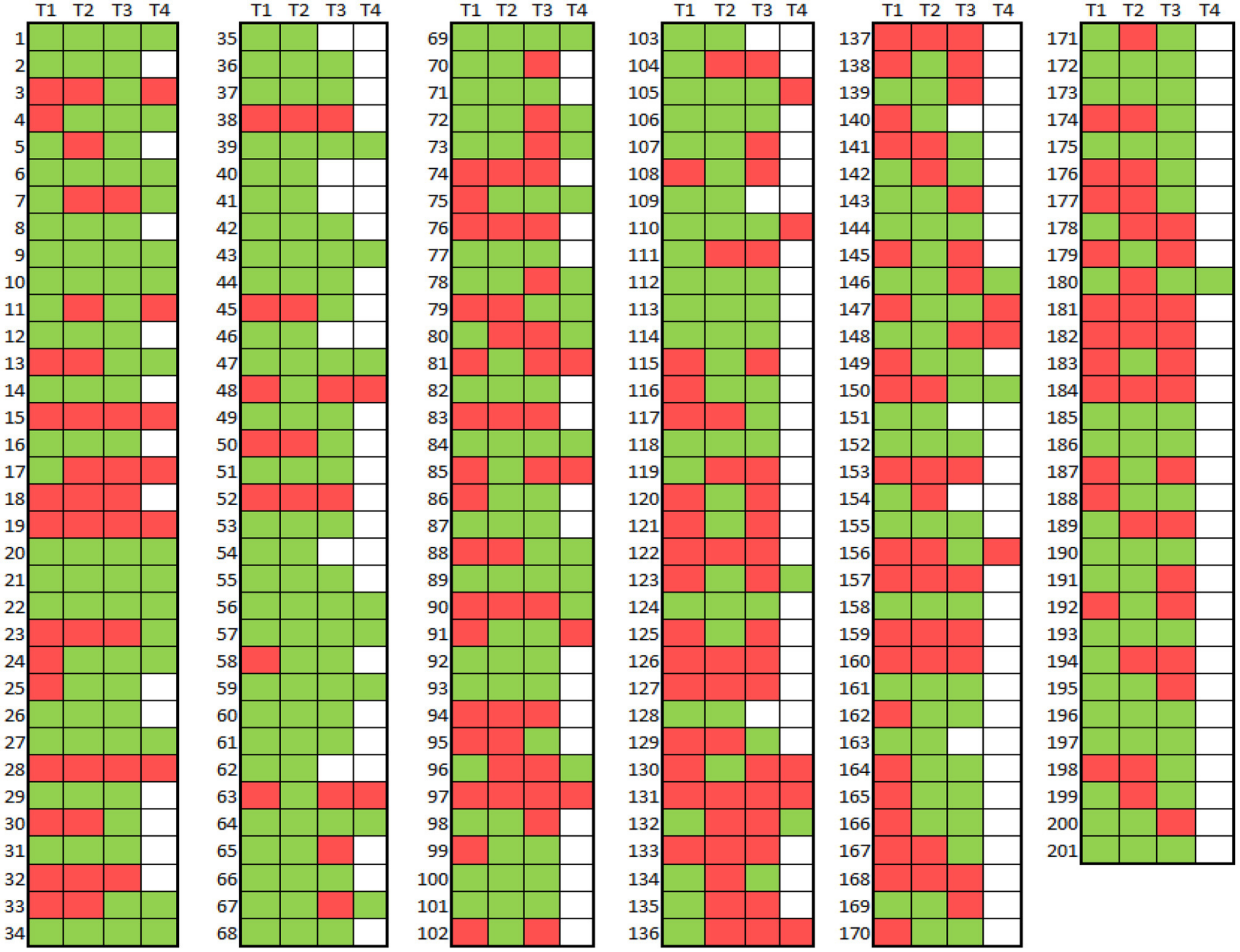
Map of ET vs. ET plus diagnoses across four time intervals in 201 participants Diagnosis = ET (red), Diagnosis = ET Plus (green). No diagnosis (i.e. no evaluation) = white.

**Figure 4:**
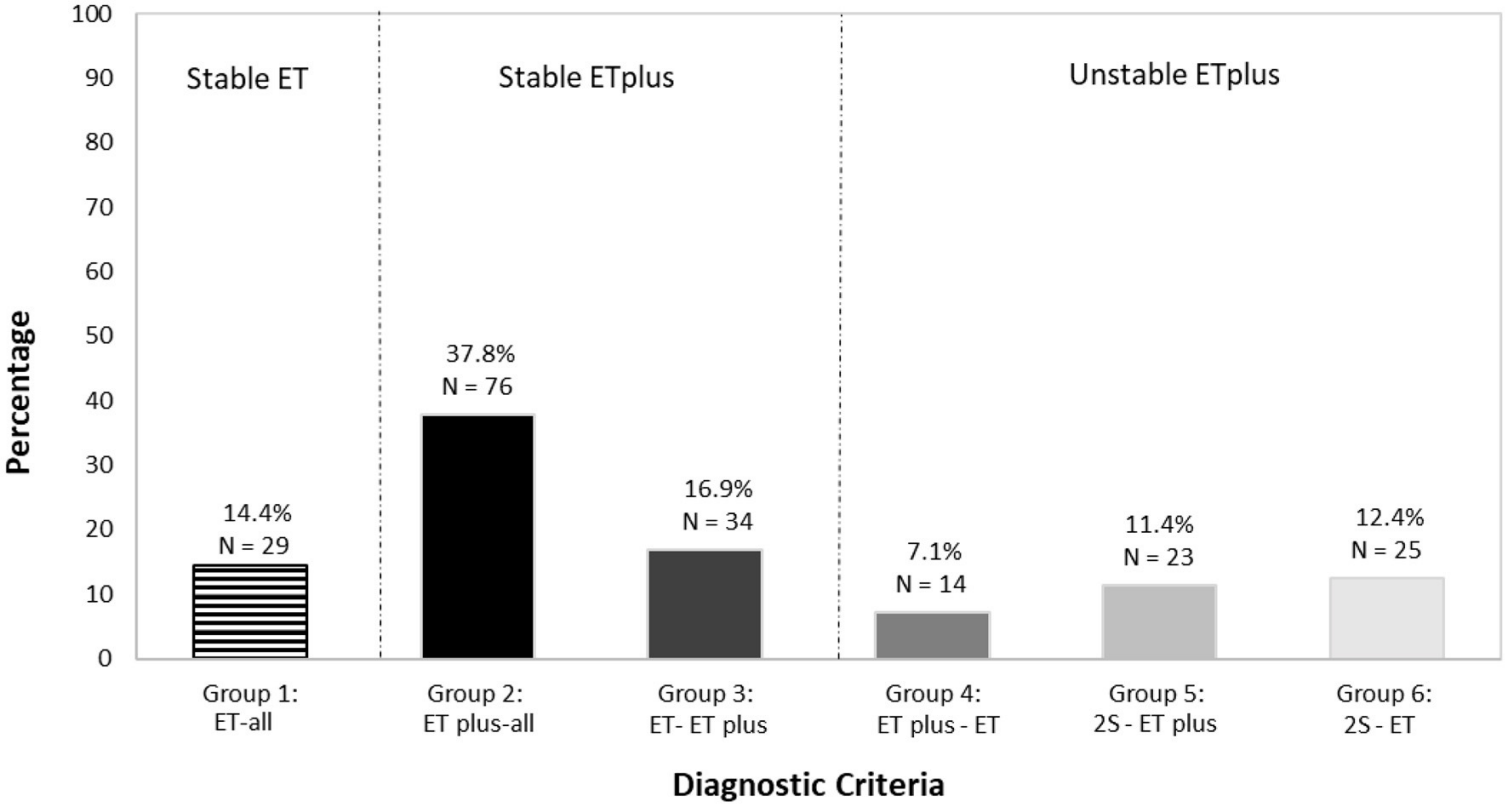
Distribution of types of ET vs. ET plus diagnoses in 201 participants.

**Figure 5:**
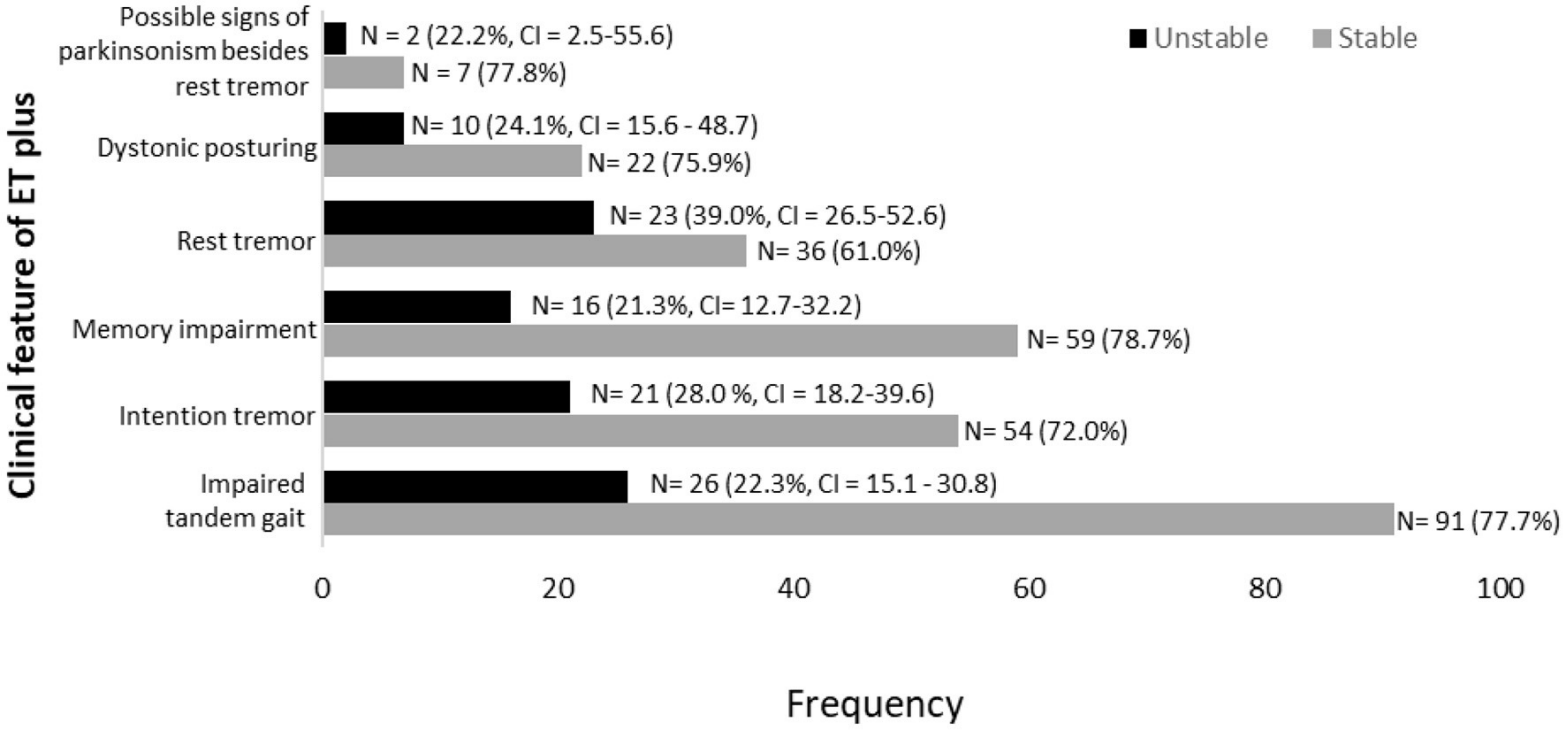
Stability of ET plus diagnostic criteria in 172 participants labeled with ET plus in at least one time interval.

SPSS v.26 was used for all statistical analyses. We assigned an ET or ET plus diagnosis to each case at each time interval. Based on the stability of the diagnosis over time, we created three “diagnostic behavior over time” categories and six “diagnostic behavior over time” groups (Figure 1). We reported the prevalence of ET vs. ET plus at each time interval (Figure 2) and developed a color-coded map of ET vs. ET plus diagnoses across four time intervals (Figure 3). Further data on the stability of ET plus diagnoses were provided by determining the proportion of each of the “diagnostic behavior over time” categories and groups (Figure 4). We performed a binary logistic regression analysis to evaluate the association between follow-up time in months and the dependent variable, ET vs. ET plus diagnosis. We assessed the stability of each ET plus diagnostic criterion (e.g., rest tremor, dystonic posturing) by reporting the proportion in which each feature reverted from present to absent at some point (Figure 5). Following the example of previous literature, we labeled these percentages as reversion rate (30). We also described the baseline demographic and clinical features of the cohort (Table 1), and compared baseline demographic and clinical features across our three “diagnostic behavior over time” categories (Table 2). We assessed the normality of each continuous variable using the Kolmogorov-Smirnov test; for normally distributed variables (e.g., age, years of education), we used one-way analysis of variance (ANOVA), and for variables that were not normally distributed (e.g., age of tremor onset, tremor duration), we used a non-parametric test (Kruskal-Wallis test) (Table 2).

**Table 1:** Baseline demographic and clinical characteristics of 201 ET cases.

**Characteristic**	**All ET cases (*n =* 201)**
Female gender	123 (61.2)
Age at baseline (years)	78.1 ± 9.5 (range = 55–96)
Education (years)	15.8 ± 2.5
Age of onset (years)	39.3 ± 22.3
Tremor duration (years)	38.5 ± 21.9
Total tremor score (range = 0 - 36)	20.2 ± 4.9
Tremor asymmetry (≥1 point)	135 (67.2)
Tremor asymmetry (≥2 points)	103 (51.2)
Currently taking medication for tremor	118 (58.7)
Number of prescription medications	5.4 ± 3.9
Cognitive diagnosis	
Normal cognition Mild cognitive impairment (MCI) Dementia Cognitive impairment related to substance abuse, trauma or stroke	159 (79) 29 (14.5) 11 (5.5) 2 (1)

*Values are number (percentage) or mean ± standard deviation*.

**Table 2:** Comparison of baseline demographic characteristics across three “diagnostic behavior over time” categories.

	**ET (*n =* 62)**	**Unstable ET plus (*n =* 52)**	**Stable ET plus (*n =* 87)**	***p*-value**
Female gender	14 (48.3)	39 (62.9)	70 (63.7)	0.39^a^
Age (years)	74.6 (9.8)	78.4 (8.7)	87.0 (9.4)	**<0.01^b^**
Education (years)	16.0 (2.4)	15.8 (2.6)	15.5 (2.4)	0.07^b^
Age of tremor onset (years)	37.7 (22.5)	37.9 (23.3)	41.3 (21.4)	0.58^c^
Tremor duration (years)	36.7 (22.6)	40.5 (20.8)	38.5 (22.1)	0.56^c^
Total tremor score	18.5 (5.1)	20.0 (5.1)	21.5 (4.3)	**<0.01^b^**
Tremor asymmetry^*^ (≥1 point)	44 (71.0)	39 (75.0)	52 (59.8)	0.14^a^
Tremor asymmetry^*^ (≥2 points)	35 (56.5)	28 (53.8)	40 (46.0)	0.41^a^
Currently taking medication for tremor	13 (44.8)	40.0 (64.5)	70.0 (63.6)	0.48^a^

*Values depicted as frequency (percentage) for categorical variables or mean (standard deviation) for continuous ones. Bolded values are statistically significant (p < 0.05)*.

*ET, essential tremor; NA, not applicable*.

* *Tremor asymmetry is defined as the difference between dominant and non-dominant arm tremor score*.

a *Chi-square test*.

b *One-way ANOVA*.

c *Kruskal Wallis-test*.

**Figure 6:**
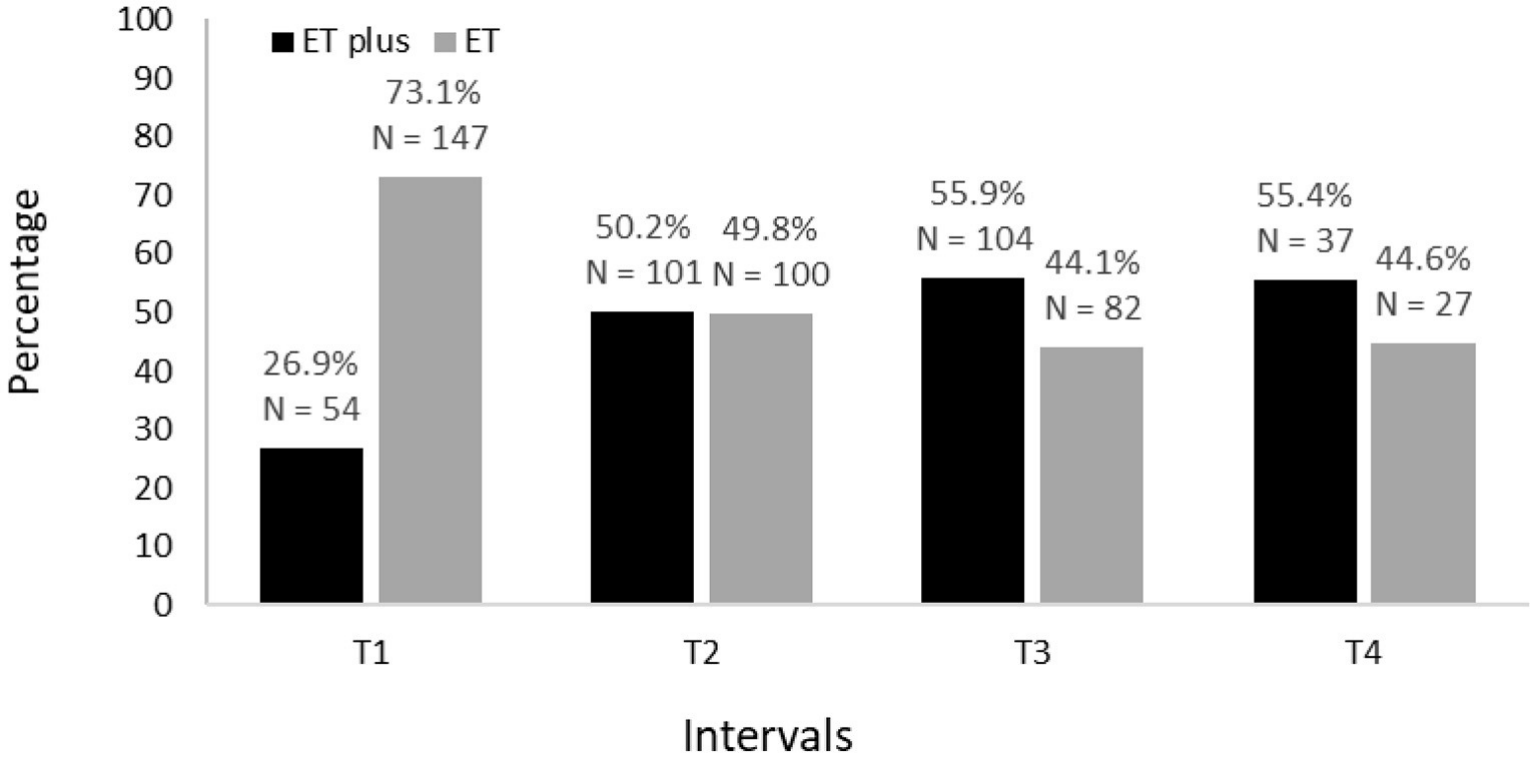
Secondary analysis of prevalence of ET vs. ET plus from baseline (T1) to 54 months after baseline (T4).

**Figure 7:**
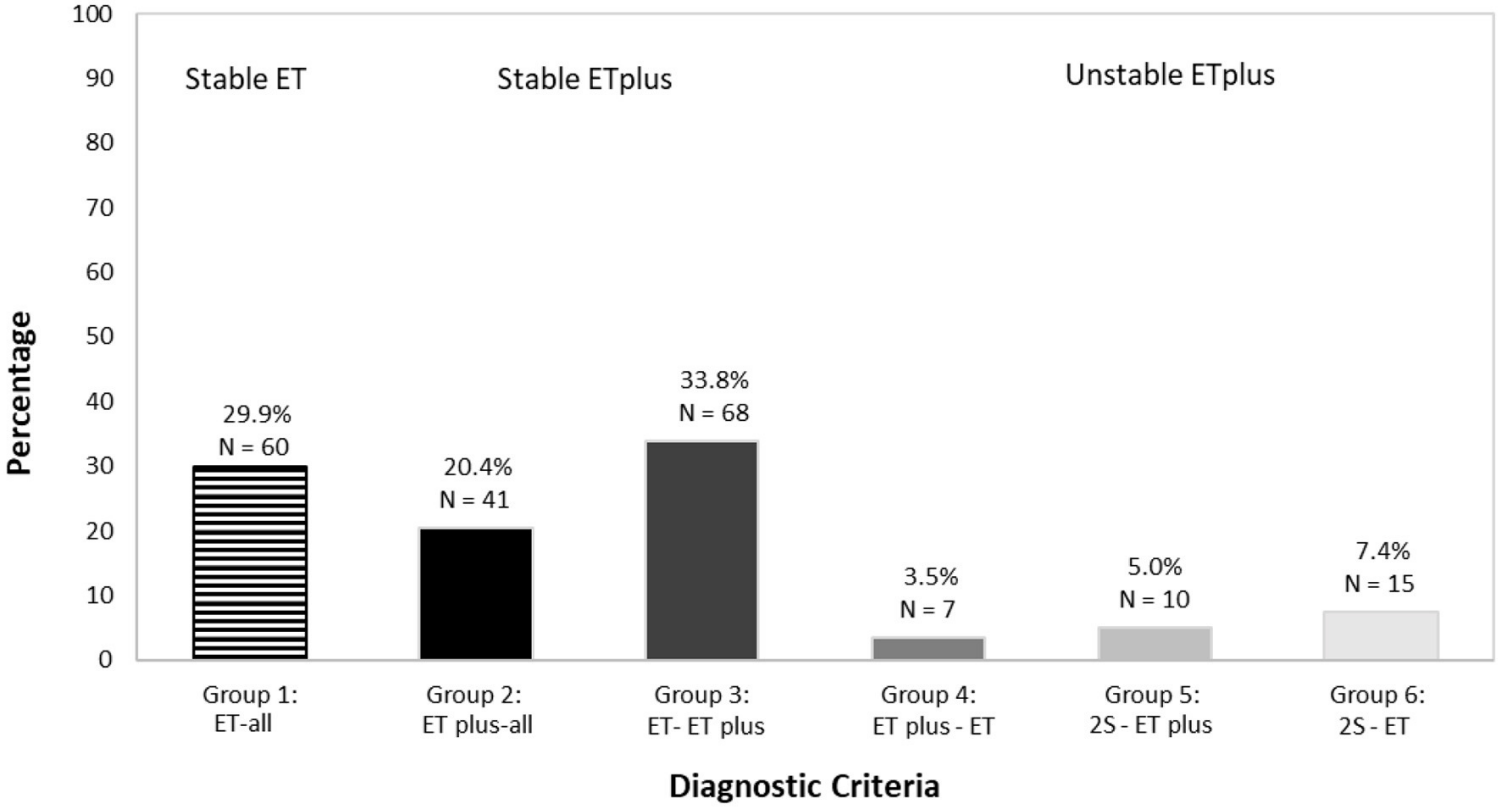
Secondary analysis of distribution of types of ET vs. ET plus in 201 participants.

In a secondary analysis, we classified each participant in the cohort but did so without including rest tremor or intention tremor as features of ET plus; we then reported the prevalence of ET vs. ET plus across time (Figure 6). As part of this secondary analysis, we also examined the frequencies of each the “diagnostic behavior over time” category and group (Figure 7).

## Results

Baseline demographic and clinical characteristics of 201 participants are shown (Table 1). The proportion diagnosed with ET and ET plus is shown for each time interval; across intervals, there is a decrease in ET diagnoses and an increase in ET plus diagnoses (Figure 2). Moreover, in a logistic regression model, longer follow-up was associated with increased likelihood of having an ET plus diagnosis (*p* = 0.046).

We mapped ET (depicted in red) and ET plus (depicted in green) diagnoses at each interval (Figure 3). There were 172 (85.6%) participants with an ET plus diagnosis at one or more time intervals. Numerous participants switched back and forth between these diagnoses across time intervals (Figure 3). Of the three “diagnostic behavior over time” categories (Figure 1), 29 (14.4%) participants were labeled ET at each time interval (stable ET diagnosis), 110 (54.7%) were stable ET plus diagnosis, and 62 (30.8%) were unstable ET plus diagnosis. Further break down of the three “diagnostic behavior over time” categories and six “diagnostic behavior over time” groups is shown (Figure 4).

Memory impairment (i.e., either MCI or dementia) was the most stable clinical feature of ET plus; it was present in 75 participants, among whom it reverted from present to absent in only 16 (21.3%) (Figure 5). Rest tremor was the most unstable clinical feature of ET plus; it was present in 59 participants, among whom it reverted from present to absent in 23 (39.0%) (Figure 5). Data for other features of ET plus are similarly shown (Figure 5). Impaired tandem gait, which was present in 117 participants, was the most prevalent ET plus feature, while intention tremor was the second most prevalent (Figure 5).

We also compared baseline demographic and clinical features across our three “diagnostic behavior over time” categories (Table 2). One-way ANOVA revealed significant differences in age (*F* = 10.6, *p* < 0.01) and total tremor score (*F* = 6.6, *p* < 0.01), with increases in age and total tremor score when comparing the ET to ET plus categories (Table 2). There was no significant difference across our groups with respect to the proportion with tremor asymmetry (Table 2). The proportion currently taking medication for tremor also differed across the categories, with the lowest value in ET. Tremor duration did not differ across the three categories, although the mean value in stable ET plus was 8 years greater than in ET.

No participant reported a diagnosis of a genetic disorder such as fragile X-associated syndrome or spinocerebellar ataxia across the four time intervals.

In a secondary analysis, we classified each participant in the cohort but did so without including rest tremor or intention tremor as features of ET plus. We still noted an increase in ET plus prevalence over time, which started at 26.9% at baseline and reached a high of 55.4% in the final time interval (Figure 6). As part of this secondary analysis, we also examined the frequencies of each the “diagnostic behavior over time” category and group (Figure 7). The proportion with unstable ET plus was 15.9%.

## Discussion

The ET plus “subtype” of ET has been the source of considerable scrutiny (10). Several cross-sectional studies have reported a prevalence of more than 50% for ET plus, after reclassifying ET cohorts into those with ET vs. those with ET plus (5, 6, 8). In the current study, 85.6% of the cohort received at least one ET plus diagnosis during the span of four time intervals and the proportion with ET plus reached 72.1% by the fourth and final time interval. Even when excluding intention and rest tremors as features of ET plus, the prevalence reached 55.4% by the final time interval, accounting for more than one-half of the cohort's diagnoses.

We tested two *a priori* hypotheses. The first was that the prevalence of ET plus would increase progressively, as it likely represents a more advanced stage of ET. Indeed, we showed that this was the case (Figure 2). The second hypothesis was that the diagnosis would not be stable over time, as cases would fluctuate with respect to their phenotypic features and their assigned diagnoses. The current study demonstrates both, with fluctuation in both assigned diagnoses (Figures 3, 4) and in phenotypic features (Figure 5). The observation that multiple cases reverted from ET plus to ET demonstrates that the “subtype” is reversible and not totally stable.

Our secondary analysis showed that 15.9% had unstable ET plus; by contrast, our primary analysis showed that this proportion was 30.9%. However, the secondary analysis involved the exclusion of rest tremor and intention tremor from the ET plus definition. As such, the secondary analysis was a departure from the Consensus statement. That statement included rest tremor with ET plus. Also, in the Consensus statement, ET plus included “mild neurological signs of unknown significance” and, in that statement, intention tremor was noted to be a neurological sign that was distinct from the type of action tremor observed in ET. As noted above, rest tremor was the most unstable clinical feature of ET plus, and intention tremor was also an unstable clinical feature in many instances. This explains the lower proportion, as noted above.

How do these results compare with those from other studies? It is impossible to make a direct comparison because there has been no prior study that has prospectively and longitudinally followed ET cases over time in order to track the changing proportions with ET vs. ET plus. The prior literature comprises cross-sectional studies that have assessed these proportions at only one point in time (5–8). Those studies show that it is possible to stratify ET cases based on clinical features. However, the above-mentioned studies do not assess the *validity* of the putative “subgroups”. One way to assess validity is to examine the stability of each putative subtype over time, as we have done here. Another way is to examine whether there are biological differences between the subgroups, as we have done previously (14).

This study should be interpreted within the context of several limitations. First, there was a decline in the number of participants over time. This decline might have preferentially affected participants with more severe disease, and while the proportion with ET plus at later time intervals was high, this decline could have led us to underestimate the proportion of ET plus at later time intervals. This would have biased us toward the null hypothesis with respect to our first hypothesis. Second, although our follow-up interval extended over many time intervals, future studies might benefit from increasing the time of follow-up (31). Third, many, though not all, of our participants were of advanced age; analyzing younger cohorts would be of additional descriptive value. Fourth, participant recruitment began prior to the introduction of the term ET plus, though this was not likely a methodological limitation since the study procedure included an extensive, systematic, detail-oriented, prospective phenotyping of all participants; therefore, high-quality, detailed data were available on each of the clinical features of ET plus (e.g., rest tremor, dystonia, cognitive performance). Last, we recognize that we made certain strategic operational research decisions, such as our stricter definition of memory or cognitive impairment. A looser definition would likely have resulted in greater interval to interval changes in the ET plus designation since some of our ET cases with minimal memory difficulty could have been classified as ET plus. Thus, our decision to incorporate a stricter definition of memory impairment biased our results toward the null hypothesis with respect to our second hypothesis.

The study possessed numerous strengths. First, the cohort was large, comprising more than 200 individuals at baseline. Second, the cohort was enrolled prospectively using a pre-defined study evaluation protocol that included an extensive, systematic, detail-oriented, prospective phenotyping of all participants. Third, the videotaped neurological examination was carefully reviewed by a senior movement disorders neurologist. Fourth, cognitive diagnoses were assigned by an expert neuropsychologist and geriatric psychiatrist using results from a comprehensive test battery. Finally, the longitudinal nature of the study allowed us to conduct the first assessment of the stability of the ET plus diagnosis over time.

In summary, these data support our two *a priori* hypotheses: (1) the prevalence of ET plus would increase progressively, as it likely represents a more advanced stage of ET, and (2) the diagnosis would not be completely stable over time, as a sizable number of cases would fluctuate with respect to their phenotypic features and their assigned diagnoses. These findings suggest that ET and ET plus may not be distinct diagnostic entities.

## Data Availability Statement

The raw data supporting the conclusions of this article will be made available by the authors, without undue reservation.

## Ethics Statement

The studies involving human participants were reviewed and approved by UT Southwestern IRB. The patients/participants provided their written informed consent to participate in this study.

## Author Contributions

DI-H: data curation, formal analysis, writing-original draft, writing-review, and editing. ND and MM: data curation, writing-review, and editing. EH and SC: conceptualization, writing-review, and editing. EL: conceptualization, formal analysis, writing-review, editing, and funding acquisition. All authors contributed to the article and approved the version submitted.

## Funding

This work was supported by the National Institutes of Health R01NS086736 and R01NS117745.

## Conflict of Interest

The authors declare that the research was conducted in the absence of any commercial or financial relationships that could be construed as a potential conflict of interest.

## Publisher's Note

All claims expressed in this article are solely those of the authors and do not necessarily represent those of their affiliated organizations, or those of the publisher, the editors and the reviewers. Any product that may be evaluated in this article, or claim that may be made by its manufacturer, is not guaranteed or endorsed by the publisher.
